# Implicit bias in referrals to relational psychological therapies: review and recommendations for mental health services

**DOI:** 10.3389/fpubh.2024.1469439

**Published:** 2025-02-07

**Authors:** Chenai Mandangu, Anne Millicent Ramos, Mohona Sengupta, Rosslyn Bender, Reem El-Hayani, Ifrah Hasan, Hannah Okechukwu, Shafeena Anas, Dominik Havsteen-Franklin

**Affiliations:** ^1^Faculty of Medicine, Imperial College London, London, United Kingdom; ^2^KCW Arts Psychotherapies Service, CNWL NHS Foundation Trust, London, United Kingdom; ^3^Team Based Learning and Education, Medical School, Brunel University of London, Uxbridge, United Kingdom; ^4^Arts and Humanities, Brunel University of London, Uxbridge, United Kingdom

**Keywords:** thematic review, implicit bias, indirect discrimination, healthcare, psychiatry, influence mapping, relational psychological therapies

## Abstract

**Introduction:**

Timely and appropriate psychological treatment is an essential element required to address the growing burden of mental health issues, which has significant implications for individuals, society, and healthcare systems. However, research indicates that implicit biases among mental health professionals may influence referral decisions, potentially leading to disparities in access to relational psychological therapies. This study investigates bias in referral practices within mental health services, identifying key themes in referral procedures and proposing recommendations to mitigate bias and promote equitable access.

**Methods:**

A systematic review of literature published between 2002 and 2022 was conducted, focusing on biases, referral practices, and relational psychological therapies. The search strategy involved full-text screening of studies meeting inclusion criteria, specifically those examining professional and organizational implicit bias in mental health referrals. Thematic synthesis was employed to analyze and categorize bias within these domains, providing a structured framework for understanding its impact on referral decision making processes.

**Results:**

The search yielded 2,964 relevant papers, of which 77 underwent full-text screening. Ultimately, eight studies met the inclusion criteria and were incorporated into the review. The analysis revealed that bias development mechanisms in referral decisions occurred across five key domains: resource allocation, organizational procedures, clinical roles, decision-making, and referral preferences. These domains highlight organizational and practitioner-level factors contributing to disparities in access to psychological therapies.

**Discussion:**

Findings suggest that implicit biases within referral processes can limit equitable access to psychological therapies, particularly relational therapies that emphasize therapeutic alliance and patient-centered care. This study provides recommendations to address these biases, including standardized referral guidelines, enhanced professional training on implicit bias, and improved oversight mechanisms within mental health services.

## Introduction

Inpatient mental healthcare professionals play a critical role in the treatment and management of individuals with mental health conditions. Referring patients to community psychological therapies after discharge is an important part of their roles and responsibilities. Existing research, such as Croskerry et al. (1), demonstrates that intuitive decision-making processes in healthcare are susceptible to cognitive biases, often leading to variability in referral quality and appropriateness.

This thematic review was conducted to investigate biases concerning patient referrals to community psychological therapies, specifically relational therapies. Healthcare professionals' perception, interpretation, and response to patient information can be influenced by biases, whether conscious or unconscious, resulting in inequitable access to suitable interventions and suboptimal treatment outcomes.

Relational therapies, which include psychodynamic therapy, person-centered therapy, attachment-based therapy, mentalisation-based therapy (MBT), interpersonal therapy (IPT), and the arts therapies (including art, music, dance movement, and drama therapy), center on therapeutic relationality as the basis for therapeutic change. These approaches emphasize empathy, trust, the co-creation of a supportive environment, and working with traumas that affect relationships as the basis of impacting symptoms produced by relational difficulties. Empirical studies [e.g., (2, 3)] suggest that relational therapies are particularly beneficial for clients from marginalized backgrounds, as these approaches prioritize the therapeutic alliance and cultural responsiveness, addressing barriers often experienced in traditional verbal therapies. For instance, arts therapies' focus on relational engagement through shared creative experiences offers an inclusive approach that goes beyond traditional verbal therapies, making it accessible to a wider range of individuals and needs (4). Further mentalization-based therapies (e.g., Mentalization Based Therapy) and interpersonal models of practice (e.g., Dynamic Interpersonal Therapy) also offer evidence-based methods of treatment for depression, and emotionally unstable personality disorder.

In contrast, cognitive and structured programmes for skills-based therapies, such as Cognitive Behavioral Therapy (CBT), Dialectical Behavior Therapy (DBT), and Acceptance and Commitment Therapy (ACT), although effective for many individuals and conditions, tend to place less emphasis on relational dynamics. CBT's structured, directive approach focuses on modifying dysfunctional thought and behavior patterns, making it particularly suitable for symptom management where there are no known relational causative factors (5). However, this comparative lack of focus on the therapeutic relationship may make CBT less suitable for clients with complex relational needs who may benefit from a more relationally engaged therapeutic modality, particularly where there has been evidence of relational trauma and abuse (6).

Excluding therapies like CBT from this review allows for a focused investigation into biases that may influence access to relational therapies specifically. This distinction is critical, as biases associated with perceptions of race, socioeconomic status, or education can restrict access to relationally focused therapies, limiting therapeutic options for marginalized groups. Such exclusions ignore evidence showing that therapeutic outcomes are often improved when clients feel respected, understood, and engaged in a relationally attuned manner, regardless of their background (3, 7, 8). By enhancing the availability of relational therapies, including arts therapies, healthcare systems can work to provide equitable, patient-centered care, promoting mental health outcomes across diverse populations (8).

While prior research has focused on biases in mental healthcare settings, there is a paucity of literature specifically examining biases in the context of referring patients to community relational therapies. Knowing the process of how these biases are produced at a macroscopic level is essential for patient-centered care and equitable access to evidence-based interventions.

This review intends to offer a more extensive comprehension of the processes that might affect the decision-making procedures of healthcare practitioners when referring patients to community relational therapies through the synthesis and analysis of relevant studies.

There are several implications resulting from the findings of this thematic review. To begin with, it will explicate the different biases that may be prevalent and how they might impact decisions made about referrals. Secondly, we shall identify the factors that influence these biases. Lastly, we will identify recommendations to improve decision-making processes.

This review aims to increase awareness of bias and therefore reduce bias and promote equitable access to community psychological therapies. Addressing biases in the referral process is critical for improving equitable access to psychological therapies.

Evidence from this review supports the implementation of structured referral criteria and targeted bias mitigation strategies to ensure that individuals with mental health conditions receive high-quality, inclusive care. In focusing this review on relational therapies, including arts therapies, the intention is to highlight how biases may limit access to these modalities.

## Background

Mental illness stands as a significant challenge within the healthcare landscape, with far-reaching consequences for individuals and society at large. In England, mental illnesses rank as the second-largest source of burden of disease (9), surpassing the prevalence of any other health condition. The impact is not only in terms of human suffering but also economically, with an estimated annual cost of £105 billion to the global economy (10). This complex challenge necessitates a comprehensive response, one that encompasses improved access to care and the reduction of biases in the referral process. The escalating prevalence of mental health disorders (11), is a concern for the development of a more inclusive society.

Further to this, Kessler et al. (11) and Wittchen et al. (41), state that less than one-third of those diagnosed with a mental illness receive any form of treatment, indicating a substantial treatment gap. This treatment gap underscores the pressing need for improved access to mental health services. The disparities in mental health prevalence among individuals from diverse protected characteristics within Western countries represent a complex challenge that amplifies the urgency of addressing mental health inequalities. Empirical evidence (12) highlights the disproportionate impact of mental ill health on specific intersections of the population. These intersections notably include individuals with disabilities, gender, as well as those within the LGBTQ+ and BAME (Black, Asian, and Minority Ethnic) communities and older people (13). Compounding these disparities is a growing body of research that points to implicit biases among healthcare providers as a central driver of unequal access to mental health services. The work of Zestcott et al. (14) has shed light on these unconscious biases, revealing how they can inadvertently lead to differential treatment and further perpetuate disparities. Implicit biases, shaped by societal stereotypes and personal experiences, operate unconsciously and significantly influence decision-making, as evidenced by Zestcott et al. (14). These biases often manifest as cognitive shortcuts that can perpetuate disparities in healthcare delivery. However, these biases are not isolated phenomena; they are influenced by personal experiences and embedded societal inequalities and stigmas (15). Merino et al. (16) identified unconscious biases in practitioner referrals, disproportionately disadvantaging vulnerable populations such as veterans, individuals experiencing homelessness, and people of color. These biases result in reduced access to appropriate mental health interventions. These biases can result in suboptimal care pathways for these individuals, ultimately hindering their access to personalized and effective mental health treatment. The strategy for the review was directly informed by the clinical backgrounds of the researchers. At the time of this research, the team consisted of a senior clinician in music therapy, a Professor of Practice in Arts Therapies, a senior lecturer from a medical school, and several undergraduate medical students. This review was conducted as part of a broader project investigating implicit bias, encompassing primary interviews, demographic data analysis, and thematic review. Each team member contributed to various aspects of the research, leveraging their unique expertise and perspectives. Their collaborative efforts were integral to each phase of the project, with roles distributed according to individual strengths and professional backgrounds to ensure a comprehensive approach to the study's objectives.

Given that referrals often hinge on practitioner discretion, a lack of insightful, collaborative and compassionate care for unprotected individuals can lead to adverse effects and impede access to services.

Considering these implicit biases in treatment decision-making, this study aims to address critical questions: What are the facilitators of implicit bias in the referral decision-making process for community psychological therapies from secondary care? Additionally, what are the individual, social, and organizational factors that facilitate implicit bias when planning care across community and inpatient services? (Table 1). By exploring these questions, this research seeks to contribute to a deeper understanding and mapping of the mechanisms through which implicit bias can affect mental healthcare decisions.

**Table 1:** PICO question development.

**Patient/population**	**Adult acute psychiatric inpatients transitioning to community mental health services**.
Intervention	Referral or lack of referral to relational psychological therapies, such as psychodynamic, interpersonal, or arts therapies.
Comparison	No referral to relational psychological therapies.
Outcome	Identification and analysis of implicit bias factors influencing referral decision-making processes, particularly in the allocation of relational therapies.

## Methods

To address the limitations posed by limited data, this review conducted a thematic analysis focused on factors influencing bias in mental health referrals. Articles published in English between 2002 and 2022 were considered. Due to limited data we aimed to examine articles over an extended period, while including papers with applicability and relevance to contemporary social advances. Extending our search to as far as 2002 reflects changes around the time relating to the promotion of consistent national guidance for treatment recommendations with the creation of NICE, with the first clinical guideline being the management of schizophrenia.

The search strategy involved four reputable electronic databases: Ovid MEDLINE, APA PsycNet, EMBASE, and EBSCOhost. To ensure comprehensive inclusion of relevant articles, a tailored search string was developed and adjusted to each database's specific syntax requirements.

This search strategy encompassed a wide range of terms related to psychological therapies, bias, and mental health, with the aim of casting a wide net for potential literature (see Table 2). We further refined the search results by limiting them to articles published in English and within the specified 20-year timeframe. Additionally, to mitigate potential publication bias, we conducted a thorough search for gray literature, encompassing exploration through Google Scholar and The King's Fund Library, both recognized sources for non-peer- reviewed materials. Furthermore, we analyzed references of articles identified through the database search to identify additional papers for potential inclusion in the review.

**Table 2:** Search terms used to identify and select papers.

**Psychological therapies**		**Implicit bias**		**Community mental health care**		**Referrals**
Psycholog^*^	AND	Bias^*^	AND	Psych^*^	AND	Referral^*^
Art therap^*^ Music therap^*^ Psychotherap^*^ Relational therap^*^ Family therap^*^ Dramatherap^*^ Dance movement therap^*^		Prejudice stereotyp^*^ Profiling Discriminat^*^ Implicit bias Social discrimination		Mental^*^ Mental disorders Secondary care Community mental health services		Transit^*^ Discharge
Psychotherap^*^		Prejudice				
		Healthcare disparities				
		Provider bias decision making				

* Was used to include alternative forms of words, plurals etc.

In this thematic review, articles were deemed eligible for inclusion if they provided descriptions of healthcare contexts wherein implicit bias had demonstrably influenced the decision-making process concerning the referral of patients to psychological therapies as part of their care planning. The inclusion criteria specified that articles must have been published since 2002 in the English language, timeframe and within Western context. We excluded articles that did not refer to a mental healthcare context or decision-making processes before or during referral processes to psychological services. The review strategy involved the identification of synonyms and alternative terms to enhance the comprehensiveness of the search. We used Boolean and proximity operators to combine the key search terms into the final search strategy.

To ensure efficient reference management, we utilized COVIDENCE software. Following the removal of duplicate records, our screening process commenced with an initial assessment of titles and abstracts against predefined inclusion and exclusion criteria (Table 3). Subsequently, full-text screening was carried out by three blinded independent assessors, adhering to the inclusion and exclusion criteria. To minimize the potential for bias affecting the selection of papers, any conflicts that arose during the screening process were resolved through discussions among all authors and the inclusion of a supervisor, collectively working to mitigate any selection bias and uphold the integrity of the review. Though no formal calibration process was used, the three assessors initially screened the first ~15% of titles and abstracts together to ensure uniformity in decision making and since the number of eligible full text papers was 77, all three assessors screened these papers independently and came to full agreement in paper selection.

**Table 3:** Inclusion and exclusion criteria.

**Inclusion**	**Exclusion**
Mention/focus within the adult inpatient care psychiatric setting and referral into the community	Focus exclusively on primary care setting
Mention/focus of potential sources or facilitators of implicit bias in the clinical decision-making process	No mention of the referral decision- making process or care planning
Papers published from 2002 onwards	Non-psychiatry or non-psychology related papers
	Articles not written in English
	Western perspective
	No access to full text articles
	No mention of a relational psychological therapy

Data from selected articles were systematically extracted, including details on study characteristics, methodologies, key findings, and insights related to implicit bias in referral decision-making for community psychological therapies. The extracted data were synthesized to identify common themes, patterns, and gaps in the literature. Given the thematic review's broad focus and lack of empirical evidence available, a formal quality assessment of included studies was not conducted. Instead, the primary focus was on mapping the existing literature and identifying areas for further research.

### Reporting

The Preferred Reporting Items for Systematic Reviews and Meta-Analyses Extension for Scoping Reviews (PRISMA-ScR) (17) guidelines were followed to ensure transparent reporting of the review process and findings.

Relevant information extracted from the included articles was systematically organized into a standardized excel form. This form was designed to capture essential details, ensuring consistency in data collection across the selected studies. The extracted information encompassed relevant elements, including author(s), year of publication, type of bias, the impact of bias, the population or context studied, and details related to the pathway process under investigation.

### Thematic analysis

The thematic analysis of the selected papers aimed to identify key themes in the referral process and propose recommendations to mitigate bias, promoting equitable access to psychological therapies. The first step in the analysis involved an immersive reading and familiarization phase. Researchers conducted an extensive review of relevant papers published from 2002 to 2022, focusing on implicit biases and referral practices within mental healthcare. This initial phase allowed the research team to gain a deep understanding of the data, noting initial patterns and insights relevant to the study's objectives (18). Following familiarization, a systematic coding process was undertaken. This step involved identifying and labeling meaningful segments, patterns, and recurring concepts within the data. The coding was adaptive and flexible, allowing new codes to emerge as the analysis progressed. This process ensured that each piece of data was categorized based on its intrinsic meaning, facilitating a structured approach to data analysis (19). After coding, the researchers developed thematic clusters representing overarching themes that transcended individual studies. These clusters provided a holistic view of the data, enabling the identification of common themes related to implicit bias in referral practices. The inductive nature of this approach ensured that the themes were derived directly from the data, enhancing the authenticity and relevance of the findings (20). A constant comparative method was employed throughout the thematic analysis. This iterative process involved continuously comparing data across studies and themes, which enhanced the rigor and validity of the analysis. This approach was crucial in ensuring that the identified themes were grounded in empirical evidence and reflected the nuances of the data (21). To synthesize the findings and develop practical recommendations, an influence map was used. This visual tool helped in understanding the relationships between themes, identifying similarities, inconsistencies, and associations within the data. The synthesis phase involved crafting a coherent narrative that conveyed the current state of knowledge on implicit bias in referral decision-making. This comprehensive overview was essential for making informed and practical recommendations.

**Figure 1:**
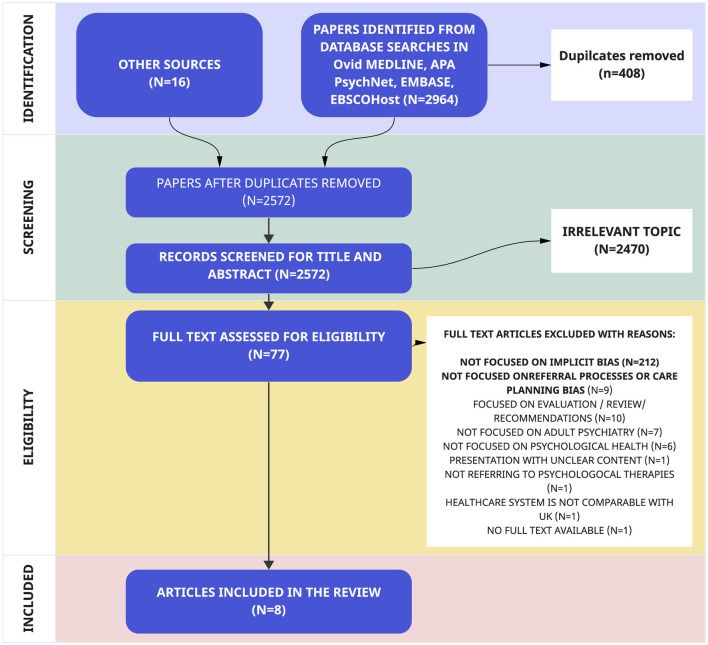
PRISMA flowchart.

The selection of articles followed the PRISMA-ScR guidelines, and the PRISMA-ScR Flowchart (Figure 1) provides a detailed account of the number of articles acquired at each stage of the review process, along with the reasons for exclusion. Out of an initial pool of 2,964 articles, 408 were identified as duplicates. Subsequently, after rigorous screening of titles and abstracts, 2,477 articles were deemed not relevant to the subject area and were therefore excluded.

Following a thorough full-text reading, an additional 69 papers were excluded as they did not meet the pre-defined inclusion criteria. Ultimately, this comprehensive selection process led to the inclusion of eight articles in the final review (Table 4).

**Table 4:** Articles included in review.

**References**	**Data collected**	**Methods employed**	**Contribution to themes**	**Identified bias**	**Primary impact of bias**	**Context/ population**	**Care pathway**	**Country**
Boswell et al. (46)	Patient- provider interaction data, referral patterns	Qualitative analysis of referral practices	Highlighted biases related to patient characteristics (e.g., verbal, compliant patients).	Bias favoring verbal, proactive patients; aversion to non-compliant patients.	Poor patient- provider match; unsystematic referrals.	Adults in community mental health centers.	Community mental health	USA
Desai et al. (26)	Interviews with Latinx and Asian adults and providers	Thematic analysis	Explored cultural stereotypes as barriers to accessing therapy.	Cultural stereotypes about Latinx and Asian groups as “less likely to benefit from therapy.”	Barriers to access for these groups.	Latinx and Asian adults and their providers.	Community mental health	USA
Fanning et al. (27)	Attendance records for group CBT sessions	Comparative quantitative and qualitative analysis	Identified educational and insight biases influencing referral success for group CBT.	Bias against patients with limited insight or education.	Barriers to completing group CBT.	Adults with first- episode psychosis.	Inpatient	Ireland
Fiddick et al. (23)	Referral decision data, clinical records	Mixed- methods: thematic and statistical analyses	Showed prioritization of physical health needs over complex psychological presentations.	Preference for patients with straightforward presentations or physical needs over complex psychological cases.	Inadequate referrals for psychological therapy.	CMHT and secondary care patients.	Community mental health	UK
Koekkoek et al. (25)	Case study reviews, practitioner interviews	Mixed- methods: qualitative and narrative analysis	Identified professional pessimism and role ambiguity in severe mental illness cases.	Bias shaped by professional pessimism and assumptions about chronicity in severe mental illness.	Increased discharge without adequate care.	Adults with severe non- psychotic illnesses.	Inpatient and Community mental health	Netherlands
Koenig (22)	Physician referral decisions, patient demographics	Quantitative survey and statistical modeling	Revealed biases based on age and race impacting treatment and referral likelihood.	Physician bias based on patient age and race influencing referral likelihood.	Under- treatment of older, racially diverse patients.	Adults aged 50+ in inpatient/community settings.	Inpatient and Community mental health	USA
Merino et al. (16)	Clinical screening and diagnosis data, practitioner surveys	Qualitative thematic analysis	Highlighted biases in clinical judgment toward marginalized populations.	Bias against marginalized populations, including veterans and homeless individuals, affecting clinical judgments.	Misdiagnose s; high healthcare costs.	Marginalized adult patients.	Inpatient and Community mental health	USA
White et al. (24)	Health professional attitudes, older adult treatment outcomes	Mixed- methods: surveys and outcome analysis	Linked stigma around mental health in older adults to reduced therapeutic engagement and suboptimal outcomes.	Stereotypes around older adults being “resistant” to therapy due to stigma.	Suboptimal therapeutic outcomes.	Older adults.	Inpatient and Community mental health	Australia

## Results

**Figure 2:**
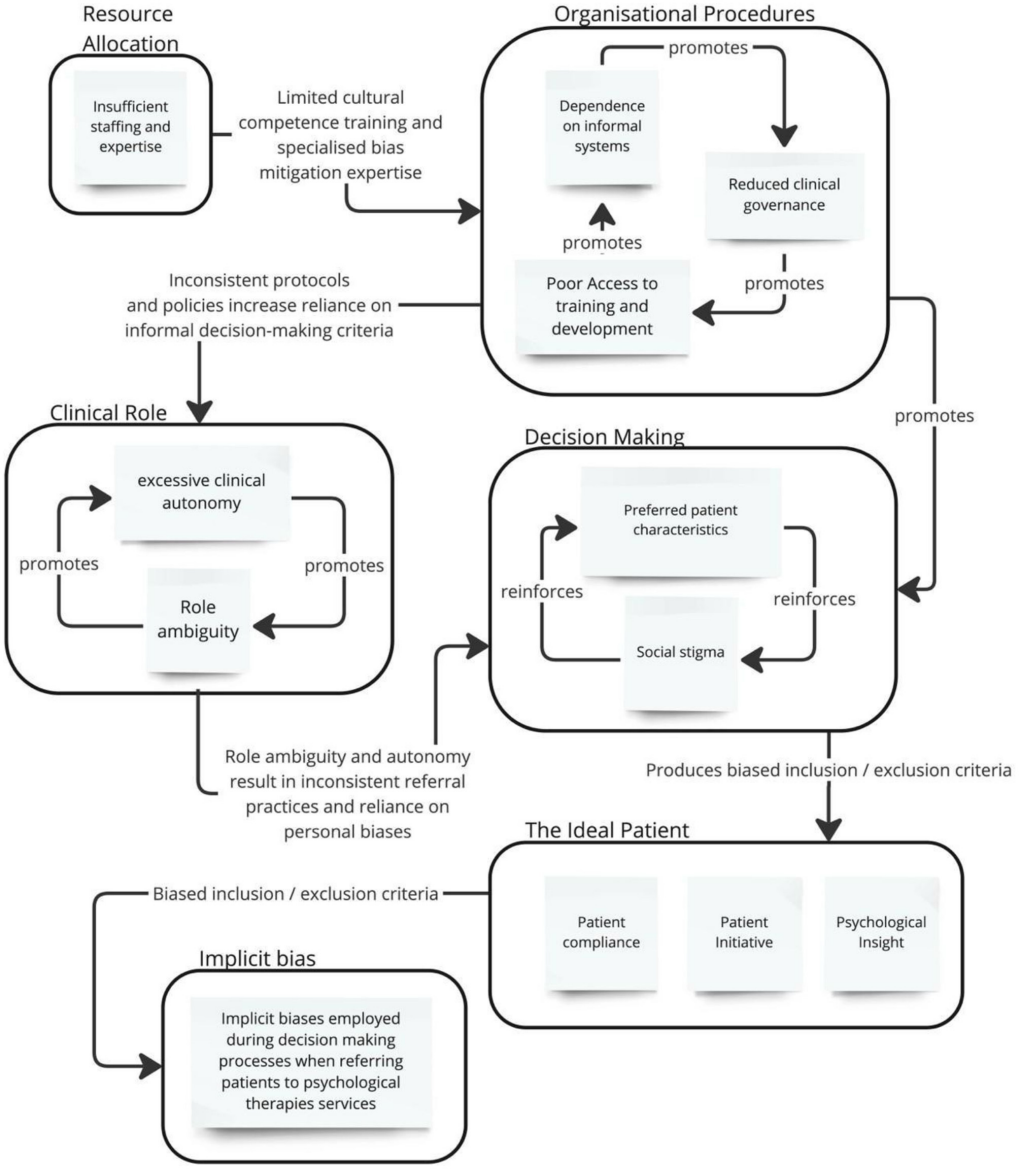
Influence map of themes and meta-themes.

We identified five overarching meta-themes and 13 themes that described contextual factors influencing the production of implicit bias within the referral processes from secondary care mental health contexts into the community (see Figure 2). The following domains where bias development mechanisms were prevalent were resource allocation, organizational procedures, clinical roles, decision-making, and referral preferences. These findings indicated that biases in referral decisions could significantly impact equitable access to mental health care. Based on the identified themes, several recommendations were proposed to mitigate implicit bias in referral practices. These include raising awareness among healthcare professionals about implicit biases, implementing standardized criteria for referrals to ensure consistency and fairness, and fostering collaborative decision-making processes that involve multiple stakeholders. These measures aim to prioritize patients' health and wellbeing, ensuring that referral practices are equitable and unbiased.

The first meta-theme, “Resource Allocation” encompasses “Insufficient staffing and expertise”, “organizational procedures” which included “dependence on informal systems”, “reduced clinical governance” and “poor Access to training and development”. The meta-theme “clinical role” included the themes of “excessive clinical autonomy” and “role ambiguity”. The meta-theme “decision making” included the themes of “preferred patient characteristics” and “social stigma”. Lastly, the meta- theme of “referral preferences” included a range of preferred patient characteristics, including “affective diagnosis disorder”, “patient compliance”, “patient engagement”, “patient initiative” and “insight”.

These meta-themes and themes provide a comprehensive framework for understanding the factors influencing implicit bias during and before the referral process, highlighting the interplay between organizational factors and professional perceptions.

### Themes

#### Resource allocation

##### Insufficient staffing and expertise

The research by Koenig (22) and Fiddick et al. (23) highlights the influence of actual and perceived resource insufficiency on implicit bias. Koenig's study indicates that in contexts where individuals face poor resource allocation or experienced perceived competition for limited resources, their implicit biases toward ethnic groups tend to increase. This is explained by the heightened need to protect limited resources, prompting individuals to resort to cognitive shortcuts like biases and stereotyping in their decision-making to manage prioritization. Fiddick et al. (23) investigate the preservation of existing dominant ideologies underpinning social and economic norms, suggesting that dominant hegemonies prevail under resource pressures. In the context of mental health care, Fiddick et al.'s (23) findings suggest that excessive caseloads and insufficient on-site resources can lead to referral decisions prioritizing physical needs and overlooking more complex and nuanced psychological and emotional patient presentations, thereby marginalizing those that do not easily fit within pre- determined schemas.

#### Organizational procedures

##### Poor access to training and development

The impact of training and professional development on implicit bias is a multifaceted theme explored within the literature. A study conducted by Merino et al. (16) suggests that when healthcare professionals receive unambiguous training on implicit bias, it has been evidenced to reduce biased attitudes and behaviors, particularly in interactions with marginalized patient populations. Such training equips individuals with the knowledge, awareness, and tools needed to recognize and challenge their biases, thereby fostering a more equitable and patient-centered approach to care. Koenig's (22) study additionally reveals age-related differences, indicating that physicians under 50 years old are more inclined to recommend counseling or psychotherapy than those over 50 suggesting that in the absence of targeted training or interventions aimed at mitigating biases, individuals, particularly those in earlier stages of professional development, may inadvertently resort to stereotypes and biases as providing normative shortcuts in their decision-making processes.

##### Dependence on informal systems

According to Fiddick et al.'s (23) research, dependence on informal systems means that practitioner decisions regarding referrals to psychological therapy are not effectively guided by formal, evidence-based criteria or patient preferences and needs. While formal systems exist to facilitate decision-making, external pressures from sources like family members and professionals outside the healthcare team can significantly sway patient prioritization. For example, a patient that has their family heavily involved in their care may sway the practitioner to carry out a referral to appease the family, though it may not be guided by formal policy or directly based on patient preferences and needs.

This introduces an element of subjective bias based on relations with the wider context, meaning that some patients are referred because the wider environment is supportive of the referral prioritization.

##### Reduced clinical governance

Reduced clinical governance, as highlighted by Merino et al. (16), poses significant challenges in healthcare. They state that one of the major problems associated with reduced clinical governance being incomplete assessments or misdiagnoses, gaps in the assessment process, leading to incomplete or inaccurate evaluations of patients' conditions. This can result in patients not receiving the appropriate care or interventions they need, exacerbated by implicit biases affecting decision-making.

When there is a lack of accountability and oversight due to clinical governance failures, Merino et al. (16) suggest that care becomes less organized, which can result in inappropriate referrals.

### Clinical role

#### Role ambiguity

White et al. (24) identifies how ambiguous roles within healthcare systems may reinforce unconscious biases, as practitioners are left without clear responsibilities or frameworks for challenging ingrained cultural norms. For example, White (ibid) highlights that there is an existing attitude that psychological comorbidities are a normal part of aging rather than something that can be treated; lack of awareness of roles within the referral process allows clinicians to lean on this attitude and may lead them to more easily overlook older adults in need of psychological therapies.

#### Excessive clinical autonomy

Merino et al.'s (16) observation that mental health services are particularly susceptible to implicit bias due to the reliance on single professionals for referral decisions raises several issues. In these settings, these professionals often function as the sole “gatekeeper” who determines which patients can access mental health services. This focus of decision-making power in one individual can create a fertile ground for implicit biases to become standard practice and influence referral choices without sufficient “checks”.

Likewise, Koekkoek et al.'s (25) research in the Netherlands highlighted the challenges that practitioners face when dealing with complex patient presentations within a system marked by excessive clinical autonomy. When practitioners were regularly tasked with making referral decisions for patients with nuanced or complex conditions, they often struggle with receiving the right support and clarity regarding referral criteria. This ambiguity was seen to exacerbate the impact of implicit biases, as practitioners resorted to normative judgments or stereotypes in the absence of clear guidelines that could provide a framework for working with differences to the norm.

### Decision making

#### Preferred patient characteristics

Desai et al.'s (26) study explores how practitioners exhibit implicit preferences for what they consider “ideal” patients, primarily driven by bureaucratic demands for efficiency. These “ideal” patients are categorized as those who are “verbal, admit a problem or illness, accept services, are proactive and individually oriented” (p. 5).

Desai et al.'s (26) study shifts the focus to implicit organizational bias within mental health treatment culture and norms. Further to this, they shed light on how these biases embedded within the system can act as barriers to engaging with diversity, favoring an idealized patient stereotype.

#### Social stigma

White et al. (24) and Koenig (22) suggested that clinicians frequently encounter patients who are resistant to engaging therapeutic interventions, primarily because they do not want to be identified as “ill” due to persistent stigma surrounding mental illness. In the context of acute care settings, these clinicians often colluded with patients' expectations that therapies would not be effective. Similarly, White et al. (24) and Koenig (22) observed that stigma associated with mental illness influenced healthcare professionals' referral decisions, often prioritizing patients who demonstrated a willingness to acknowledge their mental health needs. This suggests a need for interventions aimed at supporting patients who may avoid therapy due to stigma.

### The ideal patient

#### Patient compliance

Koekkoek et al. (25) states that healthcare professionals might develop professional pessimism when patients are labeled as “difficult” due to their non-cooperative behavior. The theme “patient compliance” reveals how mental health professionals may harbor implicit biases that lead them to disproportionately view certain patients as less likely to adhere to therapy recommendations. Koekkoek et al.'s (25) qualitative examination of the care experiences of non-psychotic long- term presentations perceived as “difficult” provides insights into how implicit biases becomes relationally enacted through healthcare professionals' perceptions, attitudes, and interactions with these patients. This theme is associated with patients being identified as challenging or not fitting normative expectations, resulting in disparities in care. These biases can stem from various factors, including demographics, clinical presentation, or past encounters with similar patients. These biases, in turn, can significantly impact referral decisions, potentially resulting in unequal access to psychological therapy services among different patient groups.

A study conducted by Fanning et al. (27) in Ireland, focusing on referrals for group Cognitive Behavioral Therapy (CBT) for first-episode psychosis (FEP), sheds light on this issue. The study analyzed patient characteristics among attendees and non- attendees of therapy sessions. The findings revealed that decisions not to refer patients were primarily influenced by perceptions of the patient's level of insight.

Interestingly, there was an assumption that a psychotic patient with impaired insight might not benefit from CBT, despite it being a recommended treatment option by NICE since 2011. Furthermore, Fiddick et al. (23) emphasized the importance of patient stability as a key factor in referral decisions, as it was seen as an indicator of the patient's readiness to engage in therapy. Additionally, the study noted that past patient experiences significantly influenced current practitioner referrals, with previous refusals making practitioners hesitant to recommend therapy again.

#### Patient initiative

The theme of “patient initiative” in the context of referrals to psychological therapy highlights the significant role of patients' proactive involvement and explicit requests for therapy in shaping healthcare professionals' referral decisions. According to Fiddick et al. (23), when patients take the initiative to request additional support for their mental health issues, it can influence healthcare practitioners' implicit biases and referral choices.

Practitioners in this context often consider a patient's request for a specific therapy as a positive sign of their willingness to engage in treatment and their potential to benefit from it. Patients' views and preferences regarding therapy play a crucial role in referral decisions. However, this preference for proactive patients can sometimes result in the de-prioritization of other patients who may be less vocal or unaware of their treatment options. Many patients in psychiatry may feel a lack of confidence in treatment, have the resources and capacity to be informed about best options or the social capital to be able to advocate for their needs.

#### Psychological insight

Fiddick et al. (23) emphasized the pivotal role of patient insight in guiding practitioner referrals, encapsulating the significance of service users' awareness of their need for psychological therapy and their willingness to initiate this change. In other words, if the patient can make sense of causative factors, narratives and diagnostic criteria, the patient is deemed as being more suitable for psychological treatment, excluding patients who are unable to make sense of their illness, who may in fact be in more need of psychological help.

### Recommendations

In response to this review, we presented the findings and consulted key NHS stakeholders about useful recommendations, considering available evidence, including reviewing existing guidelines available (28, 45).

This study employed a comprehensive strategy to generate recommendations by integrating findings from the thematic literature review with primary data collected from practitioner interviews. The insights from the thematic review formed the foundation upon which practitioner reflections could be contrasted and contextualized, setting the stage for a deeper exploration of bias in mental health services.

To build on these findings, 13 semi-structured interviews were conducted with healthcare practitioners, including psychiatric consultants, occupational therapists, nurses and psychological therapists at a London acute psychiatric service. Due to logistical limitations, a convenience sampling approach was employed over a single day, with efforts made to include representatives from each clinical staffing group to capture a wide range of perspectives. This method ensured that recommendations would reflect diverse referrer experiences within the acute psychiatric setting. Practitioner insights were analyzed using a latent inductive thematic approach, following Braun and Clarke's (18) guidelines. By focusing on latent themes, this approach allowed the study to go beyond surface-level patterns and describe into underlying beliefs, systemic challenges, and implicit factors contributing to referral biases.

The synthesis of themes from both the literature review and the interviews formed a foundation for developing targeted recommendations. By triangulating published literature with firsthand practitioner experiences, the study was able to identify alignments and gaps between theoretical and practical understandings of referral biases, enhancing the applicability of the recommendations for acute psychiatric settings. These insights were refined iteratively, incorporating feedback to ensure their relevance and practicality for clinical implementation. Through this staged process, the study produced recommendations that were both evidence-informed and attuned to the realities of clinical practice, offering a significant contribution to addressing bias in mental health referrals at both systemic and operational levels.

This process produced sixteen recommendations to reduce bias in referral processes to psychological therapies in community contexts in mental health services.

#### Increase awareness and training on implicit bias

Empirical studies [e.g., (16, 29)] suggest that targeted training on implicit bias can reduce its impact on healthcare decisions. Regular, evidence- based training programs should equip healthcare professionals with tools to recognize and address unconscious biases, fostering equitable referral practices. This training has the potential to improve professionals' self-awareness and increase their understanding of the possible biases that could impact their referral decisions (29–31).

#### Implement standardized referral procedures

Adopting structured referral pathways, underpinned by evidence-based criteria (23, 26), can help mitigate the influence of subjective judgment. Standardized protocols should prioritize patient needs and preferences while reducing reliance on practitioner discretion. The criteria should be established on evidence-based guidelines and factor in the individual needs, preferences, and characteristics of patients, rather than relying on normative cultural judgments (28, 29).

#### Foster collaborative decision-making

Collaborative approaches to care planning, including shared decision-making frameworks (28), ensure that patients' preferences and values are integral to referral decisions. Engaging multidisciplinary teams in these processes can further reduce individual bias and enhance transparency. Engage patients in the referral process by seeking their preferences and understanding their treatment aims (30, 31).

#### Monitor and evaluate referral practices

Regular audits of referral practices, including demographic analyses, can uncover trends indicative of implicit bias. For example, disparities related to race, socioeconomic status, or gender (16, 22) should inform targeted interventions and quality improvement measures. This involves scrutinizing demographic information to detect possible predispositions based on factors such as race, gender, socioeconomic status, or other pertinent factors (28, 31).

#### Promote cultural awareness and diversity

Evidence from Desai et al. (26) highlights the need for cultural competence in addressing barriers to care. Promoting diversity in healthcare teams and creating culturally responsive therapeutic environments can improve engagement and reduce biases in referrals. This includes creating environments that are welcoming and inclusive and respect and value diversity (29, 31).

#### Implement quality improvement (QI) initiatives

Introducing QI initiatives, such as feedback loops and outcome monitoring (27), can help identify implicit biases in real-time. Iterative evaluations of referral outcomes should drive continuous improvements in equity and patient-cantered care. To tackle biases in the referral process, it is advisable to introduce quality improvement initiatives such as regular audits, feedback mechanisms, and performance evaluations that assess and address the prevalent issues (28, 30).

#### Engage in continuous reflection and self-assessment

Regular reflective practices, such as debriefings and supervision (24), are essential for healthcare professionals to recognize and address personal biases. Creating safe spaces for such practices can enhance self-awareness and professional accountability. This may involve periodic discussions, debriefings, or supervision sessions to cultivate safe environments for professionals to assess their decision-making processes and challenge their biases (29, 31).

#### Support research, evidence-based interventions, and more inclusive models of psychological therapies

Future research should focus on evaluating interventions to address implicit bias and developing inclusive therapy models (4, 7). This includes investigating the effectiveness of relational and culturally tailored therapies for underserved populations. This encompasses an investigation into the efficacy of interventions that target implicit bias, as well as identifying optimal methods for enhancing the impartiality and equity of referrals to psychological treatments (28, 29).

#### Enhance data collection and usage

Comprehensive demographic data collection, as advocated by Green et al. (32), can illuminate patterns of bias in referrals. Standardizing data collection across healthcare systems ensures a robust evidence base for bias mitigation strategies. This data should include comprehensive information on race, ethnicity, gender, socioeconomic status, and other relevant factors. Analyzing this data can help identify patterns of bias and inform targeted interventions (43).

#### Implement bias mitigation tools

Utilize tools and frameworks specifically designed to reduce bias in clinical decision- making. These can include decision aids that highlight evidence-based guidelines and reduce reliance on subjective judgment. Tools like checklists and algorithms can help standardize referral processes and minimize the influence of personal biases (32).

#### Foster a culture of accountability

Embedding accountability mechanisms, such as periodic reviews of organizational policies (33), can reinforce commitments to equity. Leadership involvement is crucial in driving systemic changes and maintaining focus on bias reduction. This can involve setting specific goals for bias reduction, regularly reviewing progress, and holding staff accountable for their actions. Leadership commitment to diversity and inclusion is crucial in fostering an environment where bias mitigation is prioritized (33).

#### Engage with community stakeholders

Collaborations with community organizations can help refine referral pathways to relational therapies by addressing barriers specific to underserved populations. Williams and Mohammed (34) demonstrate the importance of community input in designing culturally relevant mental health services. By engaging community stakeholders, referral criteria can be adapted to better align with the lived experiences and needs of marginalized groups, ensuring that relational therapies such as psychodynamic, interpersonal, and arts-based therapies are accessible and resonate with diverse populations.

#### Provide tailored resources to support referral accessibility

Relational psychological therapies often require a deeper understanding of patient needs, particularly for those from underrepresented groups. Betancourt et al. (35) advocate for creating referral resources that are culturally sensitive and accessible. For instance, providing educational materials about relational therapies in multiple languages and formats can empower patients and referrers with knowledge about available options.

Ensuring accessibility for individuals with disabilities and adapting materials for varying levels of health literacy can further reduce barriers to appropriate referrals.

#### Develop anti-bias policies

Establish clear anti-bias policies that outline expectations for staff behavior and decision-making. These policies should be supported by training and resources to help staff adhere to them. Regularly reviewing and updating these policies ensures they remain effective and relevant (36). Regularly updating these policies based on evidence ensures they remain effective in promoting equitable access to relational therapies.

#### Integrate diversity in the workforce

Diversity among healthcare teams involved in referral decisions can reduce the likelihood of bias in access to relational therapies. Hunt et al. (37) argue that diverse teams bring varied perspectives, which are particularly valuable when deciding on nuanced treatments like relational psychological therapies. Recruitment efforts should aim to ensure that healthcare professionals involved in referrals reflect the diversity of the patient population, which can improve trust and communication. For example, training and mentoring programs for underrepresented clinicians in mental health can foster a more inclusive decision-making culture.

#### Implement peer review and feedback mechanisms

Structured peer review mechanisms can help address bias in referrals to relational therapies by fostering critical reflection and shared learning. Schön (44) demonstrates the efficacy of peer feedback in improving clinical decision-making. Regular case reviews that focus on the appropriateness and equity of referrals can help practitioners identify and challenge implicit biases. For example, interdisciplinary discussions about the suitability of relational therapies for patients from diverse backgrounds can refine referral practices and promote fairer allocation of resources.

By aligning these recommendations with referral processes, healthcare systems can address implicit bias and promote equitable access to relational psychological therapies. These strategies ensure that referrals are informed by patient needs and the empirical evidence supporting the efficacy of relational approaches for diverse populations.

## Discussion

The findings of this review provide crucial insights into the complex nature of implicit bias within mental health care, exploring the influences of various individual and organizational factors on implicit bias. Organizational systems may perpetuate implicit biases by relying on ambiguous referral criteria, as noted by Desai et al. (26), which prioritize efficiency over equitable access to care. Addressing these systemic flaws requires implementing standardized, evidence-based protocols.

Coupled with the often-isolated nature of healthcare work and disparities in training, this can foster an environment characterized by low accountability and transparency, making it challenging to identify and rectify implicit biases in service delivery.

Analyzing the individual processes reveals that patient and practitioner characteristics can significantly shape decision-making biases. This study highlights the disproportionate weight given to patient engagement and perceived insight, often privileging patients deemed “compliant” or “proactive” (23). Such biases can systematically disadvantage individuals who may lack the resources or confidence to advocate for their needs. Additionally, practitioners may sometimes identify certain patient groups as less than ideal, especially those in vulnerable states displaying signs of perceived non-compliance, difficulty, or aggressiveness.

The recommendations developed in this study have the potential to extend beyond mental health referral systems and impact broader aspects of patient care within physical health settings. For instance, patients admitted with conditions such as delirium secondary to infection or other physical causes may not always be recognized as candidates for specialized care, especially where there are pre-existing mental health diagnoses. From our study, there are indications that these patients can be perceived as “difficult” or “unlikely to engage,” leading to an oversight of their complex needs and potential benefits from comprehensive, multi-disciplinary support.

Addressing biases in referral pathways is essential for ensuring timely and appropriate care, particularly for patients with intersecting vulnerabilities, thereby improving holistic outcomes (14, 32).

The findings from this review suggest potential applications for addressing implicit biases in other areas of healthcare decision-making. For example, studies such as Koenig (22) have shown that biases regarding patient demographics or mental health histories can influence the allocation of resources and prioritization in physical health treatments, which may impact overall care quality.

For example, biases may influence the level of urgency, or the resources allocated to patients with mental health histories, potentially reducing the quality of care they receive for physical ailments. Integrating recommendations from this research may foster a more inclusive approach to patient assessment, encouraging healthcare practitioners to evaluate physical health needs independently of mental health backgrounds. This shift could lead to improved patient outcomes by ensuring that decisions around referrals to physical health services, like geriatrics or neurology, are driven by clear clinical indicators rather than influenced by assumptions about service engagement based on implicit biases about their mental health concerns.

Further research is needed to determine the applicability of these findings to diverse healthcare settings and patient populations. By investigating how biases in referrals manifest in various clinical contexts, future studies could provide a clearer understanding of how to implement these recommendations across specialties. This line of inquiry would support the development of training, policies, and protocols that systematically address bias, ultimately enhancing the equity and effectiveness of healthcare delivery across the spectrum of patient care. While there has been much development in the field of psychotherapies since 2007, including the introduction of digital assisted therapies and a vast increase in the number of trained psychotherapists in the UK through the Improving Access to Psychological Therapies (IAPT) NHS scheme, our findings remain relevant. The NHS aims to expand IAPT so that 1.9 million people are seen annually, though this figure accounts for just a quarter of those suffering from depression or anxiety—if demand for services and access to psychotherapies exceeds the supply, referral decisions ultimately remain at the practitioner's discretion, keeping the system flawed and susceptible to implicit bias (38).

The identified meta-theme of the ideal patient describes how implicit biases can impact healthcare professionals' perceptions and interactions with patients who don't conform to normative standards. It is arguable that a culture has developed where a lack of clear roles and systems to support collaborative work, can result in practitioners feeling compelled to align themselves with an image of the “perfect” or “ideal” patient. This alignment can reflect their own professional values and expectations, which can, in turn, impact their decisions regarding patient referrals and treatment. The complex position of being idealized as a carer, has been explored by Smith et al. (39) and deserves attention as a major contributing factor. Smith et al. [(39), p. 11] critique the code of ethics for nursing stating,

“Moreover, the expectations outlined in Section 25.1 reinforce a neoliberalized and individualist approach to the provision of nursing care; nursing is mobilized as a unidirectional commodity in service of institutional aims, foregoing any reciprocities that may exist between care-receiver and nurse care-giver.”

In other words, situated cultural nuances may be overshadowed by a highly individualistic and overly commodified form of care. Therefore, whilst we have interpreted and represented the themes present in existing literature according to clearly defined areas of inquiry, there may be a socio-political background to the problem that requires further investigation.

### Limitations

While the thematic review provides valuable insights, it is important to acknowledge its limitations. Firstly, the inclusion criteria for this study may have introduced selection bias, as studies published in languages other than English or outside the specified time frame were excluded. This could potentially limit the generalisability of the findings, and some of the more nuanced cultural perceptions of healthcare bias may have been inadvertently omitted by not including papers written in other languages.

The decision to include papers from Western settings outside the UK, such as the US, was made as there were very few papers that observed the referral decision-making process from within an inpatient psychiatric context in the UK. We felt it important to explore referring behaviors from Western countries because attitudes and stigmas toward mental health, which we have found in our review heavily influence decision making, would more closely reflect UK contexts (42).

Despite this, there are organizational and logistical differences between the mental health services across nations which means that determinants for referral may vary; for example, White et al. (24) referred to the significant impact of an inpatients ability to afford treatment on the overall decision to refer a patient onto counseling services.

This would not be widely applicable in the NHS context as most service users will be referred onto publicly funded programs.

The review's reliance on published literature introduces the potential for publication bias, as unpublished or non-English studies that may offer critical insights into implicit bias were excluded. The omission of unpublished studies could affect the comprehensiveness of the review and potentially overlook relevant findings.

The thematic review also focused specifically on implicit bias when referring patients to psychological treatments. While this provides valuable insights into this specific context, it does not address other aspects of implicit bias within mental health care, such as diagnosis, general treatment decisions, or therapeutic relationships, all of which play a complex and integral role in referrals to psychological therapies. However, the study does highlight some key themes and provides directions for future research, policy development, and practice improvement in addressing and mitigating implicit biases.

Expanding the review to encompass a broader range of healthcare environments in future studies could provide a more comprehensive understanding of implicit biases across the healthcare spectrum and offer insights into comparable healthcare contexts.

It is important to recognize that, while these recommendations are grounded in research, their effectiveness may vary in practice and could uncover additional challenges during implementation. For example, the true impact of implicit bias training on healthcare practitioner behavior has been debated in the literature, especially when such training is implemented alone without support from broader organizational changes (40). Evidence suggests that, without complementary measures—such as policy reforms, ongoing institutional support, and a culture shift—implicit bias training may have limited long-term effects on clinical decision-making (40).

To maximize the impact of these recommendations, a multi-faceted approach that combines individual training with systemic changes may be necessary. This could involve integrating bias awareness programmes with practical strategies, such as regular assessments, interdisciplinary collaboration, and continuous professional development. Such an approach would support healthcare practitioners in consistently recognizing and addressing biases across various patient care contexts, thereby fostering a more inclusive and equitable healthcare environment.

Further research into how individual bias training and organizational changes interact will be crucial to refine and validate some of the recommendations. Evaluating the outcomes of these combined approaches could provide valuable insights into creating a healthcare culture that actively supports equitable patient care across both mental and physical health contexts.

## Conclusion

In conclusion, this review underscores the pressing need for further research, policy changes, and proactive efforts in addressing implicit bias within mental health care. The identified research gaps and limitations emphasize the importance of expanding the body of knowledge in this area. Future research should aim to employ advanced methodologies, explore the intersectionality of bias, and rigorously assess the effectiveness of interventions designed to mitigate implicit bias.

On a policy level, there is a clear imperative for the implementation of measures that directly address implicit bias, promote diversity that allows for appropriate challenges of cultural and organizational norms, to ensure equitable access to mental health psychological therapies. According to the recommendations presented, these policies should be rooted in culturally and evidence informed practices and continuously evaluated to ensure their effectiveness and impact.

Additionally, healthcare professionals must play an active role in addressing and mitigating implicit bias. This includes receiving comprehensive training on recognizing and addressing bias, as well as fostering a culture of accountability and self-reflexivity within their practice. By doing so, healthcare professionals can contribute significantly to creating more inclusive and equitable mental health care environments.

## Data Availability

The dataset supporting this study is publicly available on Brunel University's Figshare repository. It can be accessed at the following link: https://doi.org/10.17633/rd.brunel.27332307.v2.
